# Desmoid tumor of the pancreas: a case report

**DOI:** 10.1186/s13256-015-0591-y

**Published:** 2015-05-06

**Authors:** Żaneta Słowik-Moczydłowska, Robert Rogulski, Anna Piotrowska, Jadwiga Małdyk, Przemysław Kluge, Andrzej Kamiński

**Affiliations:** 1 Department of Pediatric Surgery, Medical University of Warsaw, Marszałkowska 24, Warsaw, 00-576 Poland; 2 Department of Pathology, Medical University of Warsaw, Marszałkowska 24, Warsaw, 00-576 Poland; 3 Department of Pathology, The Children’s Memorial Health Institute, Al. Dzieci Polskich 20, Warsaw, 04-730 Poland

**Keywords:** Desmoid tumor of the pancreas, Fibromatosis, Beta-catenin, Pancreatic tumor, Familial adenomatous polyposis

## Abstract

**Introduction:**

Desmoid tumor is a rare, benign, usually asymptomatic fibromatous lesion. The etiology is unknown and the diagnosis is based on histopathological examination. The treatment is complete resection of the tumor. Pancreatic desmoid tumor is extremely rare. In the literature there have been only 11 cases described, most of them as solid or solid-cystic masses. We report the case of a patient with an isolated cystic pancreatic desmoid tumor that is, to the best of our knowledge, the second reported case.

**Case presentation:**

A 13-year old Caucasian boy presented with recurrent pain of two months’ duration in the left hypochondrium of his abdomen. An ultrasound examination and computed tomography scan revealed the presence of a cystic mass located in his splenic hilum, tightly adjacent to the pancreatic tail. A splenic cyst was suspected. Operative findings showed a 10x10cm cystic mass tightly connected to the pancreatic tail and left colonic flexure, adherent to the spleen, splenic vein and artery. Distal splenopancreatectomy with *en bloc* resection of the left colonic flexure was performed. Histological analysis confirmed that the resection was complete. The mass had infiltrated the pancreatic parenchyma. All tumor cells were positive for anti-beta-catenin staining characteristic for desmoid tumor. No abnormalities in the spleen and colon were found.

**Conclusions:**

Isolated sporadic pancreatic desmoid tumor with cyst formation is extremely rare and its diagnosis can be difficult, especially because of uncharacteristic symptoms and radiological findings, as in our patient. This case report should be of interest not only to surgeons, as the treatment of choice is radical resection, but also gastroenterologists, considering it is in close relation with familial adenomatous polyposis, and oncologists as the reason for differentiation with other pancreatic tumors.

## Introduction

Desmoid tumor (DT) is a particular type of fibromatosis. It is a rare fibromatous lesion that is a result of abnormal proliferation of myofibroblasts. DT generally involves fascial and musculoaponeurotic tissues. It comprises 0.03% of all tumors and 3% of fibrous tissue tumors [1,2]. We can distinguish DT of abdominal or pelvic wall - 49%, extra-abdominal DT (shoulder girdle, chest and inguinal region) - 43% and intra-abdominal DT (retroperitoneum and mesentery) - 8% [3]. Intra-abdominal DTs are often observed in familial adenomatous polyposis (FAP) - concerning about 30% of these patients [4,5]. Desmoid tumors are benign, do not metastasize, but their advancement can be life-threatening due to aggressive local invasion. DTs usually are asymptomatic for a long period of time before the diagnosis. The etiology remains unclear. Attention is paid to the role of injury (postoperative scars), hormonal (estrogen - higher incidence of DT in women during their reproductive years, pregnancy, disappearance after menopause, regression under the influence of tamoxifen) and genetic factors (elements of FAP or Gardner’s syndrome - *APC* gene mutation) [1,3,6]. The diagnosis is based on histopathological examination that shows fibroblastic proliferation confirmed with positive beta-catenin immunohistochemical staining. There is no clear statement concerning the treatment. The most effective method seems to be complete resection but considering the high tendency of recurrence, complementary treatment such as tamoxifen, cytotoxic chemotherapy and radiotherapy still constitutes a research subject [1].

Isolated pancreatic DT is extremely rare. In the literature there have been only 11 cases described of pancreatic DT with eight located in the tail. Seven out of 11 published cases were solid mass, with only three solid-cystic and one cystic mass [3,4,6,7].

We report the case of a patient with an isolated cystic mass located in the pancreatic tail diagnosed by beta-catenin immunohistochemical staining as a DT; it is, to the best of our knowledge, the second reported case.

## Case presentation

**Figure 1:**
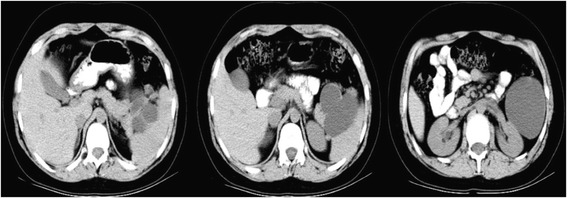
A computed tomography scan showing a cystic mass (10x7x9cm) with multiple septums located in the splenic hilum, tightly adjacent to the pancreatic tail and the left kidney.

**Figure 2:**
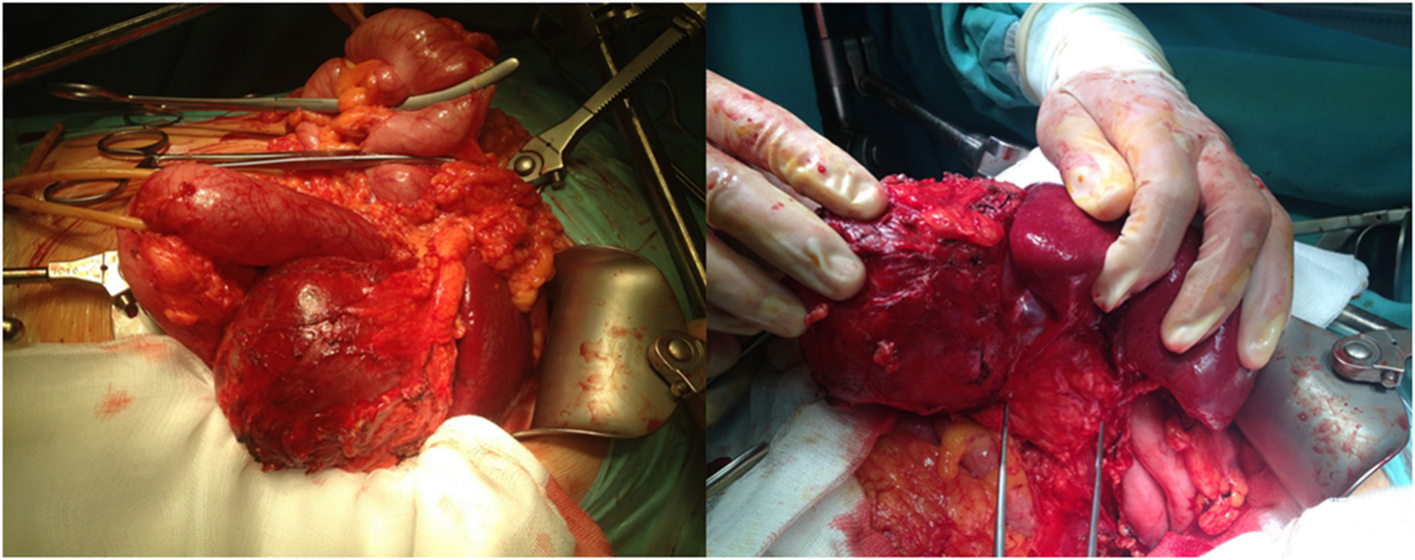
Intraoperative findings: a 10x10cm cystic mass tightly connected to the pancreatic tail and left colonic flexure, adherent to the spleen, splenic vein and artery, but not the stomach and left kidney.

**Figure 3:**
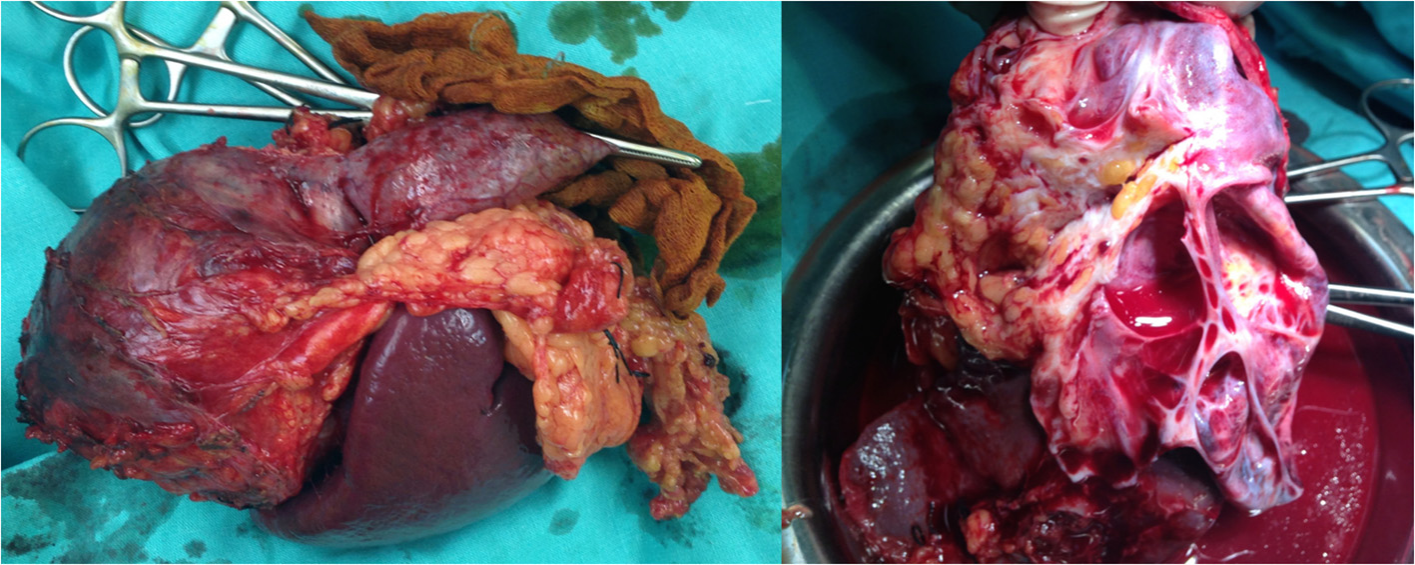
Distal splenopancreatectomy with *en bloc* resection of the left colonic flexure (on the left). A well-circumscribed cyst with a thick wall and multiple septums, filled with white fluid (on the right).

**Figure 4:**
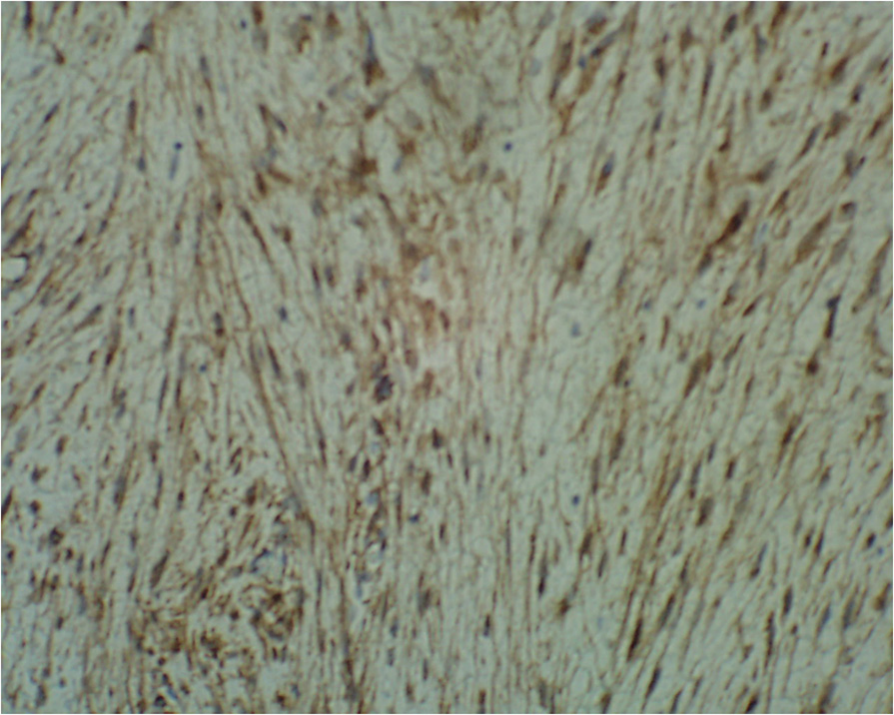
Immunohistochemical staining: tumor cells positive for anti-beta-catenin x400.

A 13-year-old Caucasian boy presented to our department with recurrent pain in the left hypochondrium of his abdomen of two months’ duration. Laboratory tests showed no abnormalities. An ultrasound examination and computed tomography (CT) scan revealed the presence of a cystic mass (10x7x9cm) with multiple septums located in his splenic hilum, tightly adjacent to the pancreatic tail and the left kidney (Figure 1). A splenic cyst was suspected. Test results for *Echinococcus* antibodies were negative. Our patient was qualified for radical surgery. Before the operation, the patient was vaccinated with pneumococcal and meningococcal vaccines as recommended in cases of elective splenectomy. Operative findings showed a 10x10cm cystic mass with a thick, grey white wall, tightly connected and macroscopically invading his pancreatic tail and left colonic flexure, spleen, splenic vein and artery, but not his stomach and left kidney (Figure 2). Distal splenopancreatectomy with *en bloc* resection of the left colonic flexure was performed as a guarantee of radical tumor excision. The macroscopic examination revealed a well-circumscribed cyst with a thick wall and multiple septums, filled with white fluid (Figure 3). Histological analysis confirmed that the resection was complete. The mass had infiltrated the pancreatic parenchyma and consisted of fibroblastic cells with a regular nucleus with low mitotic activity, with abundant extracellular collagen matrix. The pancreatic tail ducts were dilated with nonspecific inflammation and calcification in its wall. All tumor cells were positive for anti-beta-catenin staining (Figure 4). No abnormalities or DT cells were found in the spleen and colon.

Postoperative course was complicated by pancreatic leakage successfully treated with endoscopic retrograde cholangiopancreatography with Wirsung’s duct stenting. Because of the complete resection and sporadic origin of the tumor, no corresponding treatment was given. Bearing in mind that intra-abdominal DTs are commonly connected with FAP, our patient underwent colonoscopy, which revealed no changes in the colon wall.

After 19 months of follow-up, the clinical examination and ultrasonography (USG) are normal and show no recurrence of the DT.

## Discussion

Desmoid tumor is a very rare fibromatous lesion, a result of abnormal myofibroblast proliferation. DTs are benign, do not metastasize but can be locally aggressive. Localization of DTs can be abdominal or pelvic, extra-abdominal or intra-abdominal. Intra-abdominal DTs are the rarest and mostly associated with FAP. Pancreatic DTs, as described in our patient, are extremely rare, are mostly located in the tail of the pancreas, and occur as a solid mass. To the best of our knowledge, only three cases from those already reported were with a cystic component and only one case occurred as a cyst like in our patient. DTs are usually asymptomatic for a long period of time or can cause uncharacteristic pain as observed in our case. An initial diagnosis of DT based on radiological findings and laboratory tests is impossible. It requires differentiation from other abdominal cystic tumors. The final diagnosis can be based only on histological and immunohistochemical findings [8]. In our case, our patient was qualified for surgery while splenic cyst was suspected. The diagnosis of DT was unexpected and confirmed by positive nuclear immunostaining of beta-catenin. The treatment of choice for DT seems to be a complete resection with clear margins. However, many reports suggest, with benign tumors such as DT, complementary treatment with tamoxifen, chemo- or radiotherapy [1]. Also successful treatment with nonsteroidal anti-inflammatory drugs has been reported [9]. In our case, distal splenopancreatectomy with *en bloc* resection of the left colonic flexure with histopathologically confirmed uninvolved margins was the reason for no complementary therapy. Our patient remains disease-free 19 months after the surgery. Taking into account the close relationship between DTs and FAP, investigations for colonic changes should be undertaken. In our case, colonoscopy revealed no changes in the colonic wall.

## Conclusions

Isolated sporadic pancreatic desmoid tumor with cyst formation is extremely rare and its diagnosis can be difficult, such as in our case, where it preoperatively presented as a splenic cyst. Even though DTs are benign and do not metastasize, they can be locally aggressive. It is considered that when possible, the treatment of choice for DT is radical resection with clear margins, as in our patient.

## Consent

Written informed consent was obtained from the patient’s legal guardian(s) for publication of this case report and any accompanying images. A copy of the written consent is available for review by the Editor-in-Chief of this journal.

## Abbreviations

CT: computed tomography
DT: desmoid tumor
FAP: familial adenomatous polyposis
USG: ultrasonography
